# Understanding gender norms, nutrition, and physical activity in adolescent girls: a scoping review

**DOI:** 10.1186/s12966-015-0166-8

**Published:** 2015-01-24

**Authors:** Rebecca A Spencer, Laurene Rehman, Sara FL Kirk

**Affiliations:** Applied Research Collaborations for Health, Dalhousie University, 1318 Robie St, Halifax, NS B3H4R2 Canada; School of Health and Human Performance, Dalhousie University, 6230 South St, Halifax, NS B3H4R2 Canada

**Keywords:** Gender norms, Physical activity, Nutrition, Adolescent girls, Scoping study

## Abstract

Public health is currently focused on childhood obesity, and the associated behaviors of physical activity and nutrition. Canadian youth are insufficiently active and do not meet nutritional guidelines. This is of particular concern for adolescent girls, as they are less active than boys, become less active as they age, and engage in unhealthy weight control behaviors. The purpose of this review is to determine what is known from the existing literature about how gender norms are understood in relation to the health-related behaviors of PA and nutrition in young girls. This scoping review follows the framework of Arksey and O’Malley, involving defining a research question, study identification and selection, charting, interpretation, summarizing, and reporting. In total, 28 documents are reviewed, and characteristics are summarized quantitatively and qualitatively. Five major themes are identified: (1) Girls’ relationships with PA are complex and require negotiating gender roles, (2) the literature focuses on dieting rather than nutrition, (3) appearance and perceptions influence behaviors, (4) “body” focused discourse is significant to girls’ experiences, and (5) social influences, institutions, and environments are influential and may offer opportunity for future research and action. Gaps in the literature are identified and discussed. It is concluded that young girls’ activity and nutrition is affected by gender norms and feminine ideals through complex negotiations, perceptions, body-centered discourse, and societal influences.

Public health is currently focused on childhood obesity, with 32% of Canadian youth being overweight or obese [1]. In 2006, the direct cost of childhood and adult obesity was an estimated 4% of healthcare expenditures in Canada, a figure that does not include indirect costs, and that is expected to increase alongside rates of obesity [2]. Nutrition and physical activity (PA) are two potentially contributing factors to obesity, and while often looked at as individual behaviors, the population health perspective is increasingly recognizing that broader factors, including obesogenic environments, contribute greatly to the health of individuals [3]. Canadian children and youth are not receiving adequate nutrition, particularly in regard to fruit and vegetable consumption [4,5]. Similarly, fewer than 7% of Canadian youth achieve the Canadian Society for Exercise Physiology’s guidelines of 60 minutes of daily PA [6]. Canadian girls are less active than boys, with only 4% meeting PA recommendations, compared to 9% of boys [6]. Over the lifespan, participation in PA declines, and this decline is greater for girls [7].

While it is understood that gender norms contribute to health behaviors, little research has been conducted exploring the relationship between gender norms, PA, and nutrition in young girls. For this paper, young girls were defined as those aged 10–19, in accordance with the World Health Organization’s definition of adolescence; gender norms were defined as the socially constructed and accepted roles and stereotypes ascribed to gender. The purpose of this paper was to determine what is known from the existing literature about how gender norms are understood in relation to the health-related behaviors of PA and nutrition in young girls.

## Review

### Methods

This review took the approach of a scoping study, originally described by Arksey and O’Malley and updated by others [8-10]. Scoping studies address broad areas of evidence, exploring breadth rather than depth, and generally do not assess quality [10]. They are appropriate for understudied, complex areas, and may include examining the available evidence or identifying gaps in the literature, making the scoping study relevant for this review [10,11]. Arksey and O’Malley (2005) described the scoping study as a 6-stage framework, outlined as applied in this study in Table 1 [10].

**Table 1:** **Search method, following six-stage framework outlined by Arksey and O’Malley (2005)**

**Stage 1:** Defining the research question	For this review, the research question was, “What is known from the existing literature about how gender norms are understood in relation to the health-related behaviors of PA and nutrition in young girls?”
**Stage 2:** Identifying all relevant studies	The majority of literature was identified through database searching, which was followed by hand searching of reference lists, key journals, and authors [10]. Relevant networks were also contacted, such as a local initiative promoting girls’ health, to identify further evidence. A meeting was held with a university library subject specialist to identify a search strategy for the databases EMbase, PubMed, and EBSCOHost. The library specialist helped identify indexed subject headings: the concept of PA, for example, is identified by “exercise”, “physical fitness”, and “sports”, dependent on the database. Key word search strategies were developed, including “gender”, “physical activity”, “nutrition”, and “girls”. Variations for each keyword were combined with the “OR” operator to maximize results. The search function ‘AND’ was used to identify articles with a focus on both nutrition and PA, although articles with either PA or nutrition were also retrieved.
**Stage 3:** Study selection	An iterative process was used to establish inclusion and exclusion criteria as an understanding of the literature developed [10,11]. Inclusion criteria involved limiting the search to include English articles published since January 1, 1999 involving female adolescents aged 10–19 years. The most important criteria were that documents included examination of gender norms, roles, ideals, or stereotypes, and were in some way were connected to PA and/or nutrition. To ensure included articles focused on the sociocultural construct of gender norms, studies were excluded if they were centered on sex rather than gender, medicine, biology, physiology, or basic science. Adhering to the method of Arksey and O’Malley (2005), quality assessment is beyond the scope of this study.
**Stage 4:** Interpreting and synthesising	Data were interpreted and synthesized through charting the data. A narrative approach was used to create a chart for study characteristics such as year, place, participants, objectives, methods, measures, and results (summarized in Table 2) [10].
**Stage 5:** Summarizing and reporting	Results were summarized in order to present an overview of the evidence [10,11]. Quantitative and qualitative analyses were used to describe study characteristics [10]. These analyses allowed major themes and gaps in the literature to be identified, through noting commonality across the chart described above, the results of which are described further below [10].
**Stage 6:** Consultation	This stage, considered optional, allows involving experts in the review process and, for this study, was incorporated during stage 2, as it made sense to engage organizations in the search process [10]. A briefing note summarizing the review for policy makers furthered this stage as an act of knowledge translation.

### Search outcome

**Figure 1:**
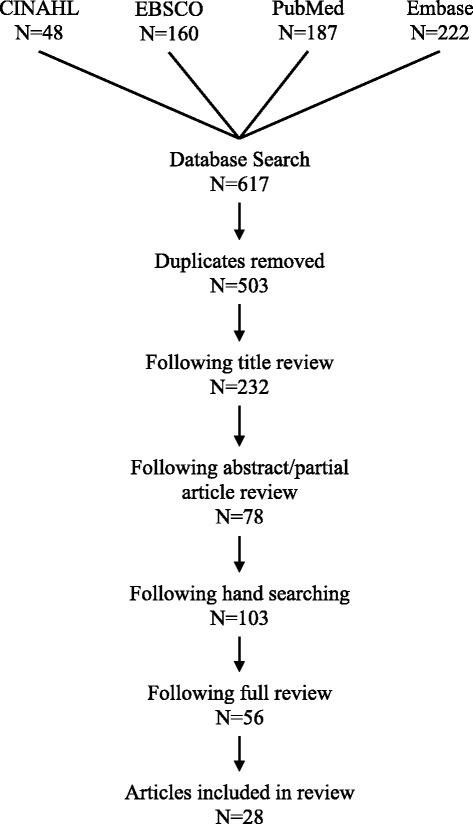
**Search outcome.**

The database search strategy was executed, duplicates were removed, and titles were reviewed in order to exclude articles not connected to the review’s purpose. The iterative process of excluding articles involved reviewing and reading at progressively more thorough levels (titles, then abstracts, then full papers), in order to apply inclusion/exclusion criteria, resulting in 28 articles being included. Two graduate students assisted in reviewing articles. Figure 1 further describes this process. A charting process was used to extract data from each article, which is summarized in Table 2. Quantitative characteristics of reviewed studies, such as article type, populations, and settings are summarized in Table 3.

**Table 2:** **Summary of charting process (Stage 4)**

**Authors year; Country**	**Objective/Aim**	**Population**	**Design, Methodology**	**Key points**
Azzarito L, Solmon MA, Harrison L. 2006; UK	To explore gender roles in PA with postructuralism, investigating how girls negotiate gender relations in PE classes.	High school PE class: teacher and 15 girls, diverse population	Discourse analysis and feminist poststructuralism; Interviews with teacher and girls, field notes, observation.	Evidence supporting complexity of gender barriers; Girls need support in negotiating stereotypes.
Barr-Anderson DJ, Neumark-Sztainer D, Schmitz KH, et al. 2008; USA	To use a socioecological framework in examining factors influencing PE enjoyment.	6th grade girls (n = 1511), diverse population	Part of the Trial of Activity for Adolescent Girls (TAAG) to promote girls’ PA; Survey (self-efficacy, perceived benefits of PA), BMI, PA.	PE enjoyment high; Found inverse relationship between BMI and PE enjoyment; Teacher influence associated with PE enjoyment.
Brooks F, Magnusson J2007; UK	To explore how active adolescent women experience leisure PA, and their perceptions of the relationship between PA and health.	Adolescents aged 14–16 (n = 42), including 6 males	Part of larger study of youth PA in UK health centers; 7 focus groups (2 all girl, 5 mixed gender).	Girls expressed enjoyment of leisure PA but disliked competitiveness of sports; Found PA can be empowering experience, perceived positively for health and wellbeing.
Clark MI, Spence JC, Holt NL 2011; Canada	To understand how adolescent girls experience PA in their daily lives.	Girls in 6th grade (n = 8), from 1 school in Western Canada	Interpretive description. Two interviews; pre-interview activities including creating collages or drawings.	Found girls have complex relationships with PA. Recommended interventions consider creating spaces where girls feel empowered to negotiate experiences.
Cockburn C, Clarke G 2002; UK	To explore the aspects of young girls' lives that influence involvement in sport and PE and identity formation.	Girls in 9th grade (n = 6); unspecified ethnicity	Reflexive interpretation and biographical analysis; in-depth semi-structured interviews.	Girls PA can be interpreted as resistance to dominant forces. Found girls felt need to compensate for deviation, creating ‘femininity deficit'.
Coller TG, Neumark-Sztainer D. 1999; USA	To develop, implement, and evaluate an after-school program to prevent weight occupation and dieting.	Girls ages 10–12 (n = 22); unspecified ethnicity	Intervention in Girl Scout program to address attitudes toward eating, PA, body image, and weight control; Surveys.	Found minimal change in attitudes and behaviors; Concluded intervention needed better focus and that future programs should explore influence of media.
Derenne JL, Beresin EV2006; USA	To explore and explain the historical context of eating disorders in relation to body image and the media.	N/A: Commentary	Commentary	Explained female ideals change over time but have been unrealistic, associated with fertility, femininity; Current media sends mixed messages about what is attractive; impacts health.
DeRose LF, Das M, Millman SR 2000; USA	To conduct a review exploring how gender bias affects food distribution.	N/A: Literature review	Literature Review	Indicates women are not at a disadvantage in food allocation; Suggest results difficult to interpret.
Dunkley TL, Wertheim EH, Paxton SJ 2001; Australia	To test relationships and mechanisms of sociocultural variables in exploring role of media, parents, peers on body image and dietary restraint.	Girls in 10th grade (n = 577) from 6 schools.	Surveys/ scales (pressures from parents, peers, media, body image, and dietary restriction).	Only 15% said they never watched their weight, 30% said they always did. Combined influence of media, parents, and peers was greatest on body dissatisfaction and dietary restraint. Best predictor of body dissatisfaction was larger body size.
Eisenberg ME, Neumark-Sztainer D, Story M, Perry C 2005; USA	To examine relationship between two social factors (girls trying to lose weight and perceptions of friends dieting), and unhealthy weight control behaviors.	Junior and senior high school girls from 29 schools (n = 2337), from ethnically diverse community.	Part of Project EAT (Eating Among Teens); Survey (dieting norms), BMI.	Found social norms from peer group and at school level may influence UWCBs, particularly for average weight girls.
Evans B 2006; UK	To compare experiences of girls-only PE to mixed gender PE, using theory of corporeal femininities and inhibited intentionality.	Female adolescents aged 13–16, 90% white.	Draws on data from qualitative and quantitative research with adolescents in UK.	Noted pressure to be both feminine and good at sport; Suggest providing choice and promoting enjoyment, as well as education about resisting pressure to conform to ideals.
Ferrar KE, Olds TS, Walters JL 2011; Australia	To explore gender-specific time use patterns in adolescents.	Australians aged 9–16 (n = 2200); unspecified ethnicity.	Data from National Children's Nutrition and Physical Activity Survey; Analysis of 24-hour time use recalls (n = 8800).	Found boys spent more time in PA, girls spent more time socializing and studying; Suggest gender differences reinforce stereotypes.
Flintoff A, Scraton S.2001; UK	To explore young women’s perceptions of and attitudes toward PA and PE.	Girls age 15 (n = 21) from 4 schools in multi-ethnic community.	Draws on data from a study of young people's lifestyles using feminist theory. Group and individual interviews.	Found girls identified positively with PA, but disliked how PA structured. Noted support from peers/ teachers important and need for review of PE delivery.
Guendouzi J 2004; USA	To examine extracts from women's conversations, exploring social pressure to conform to acceptable body size.	Women teachers and teenage girls.	Discourse analysis and discursive psychology. Audio taped conversation by teachers during breaks, and teenage girls at weekly gatherings.	Noted women have complex relationship with their bodies, and both help create and reproduce thin ideals.
Heywood 2003; USA	Book chapter about author’s own female teen experiences with PA.	N/A: Book chapter	N/A: Book chapter	Notes attaining ideals is impossible, and how knowing and understanding this is important. States need for societal redefinition of gender roles.
Holman MJ, Johnson J, Lucier M-K 2013; Canada	To explore link between body-based harassment and girls' adoption of healthy choices (eating habits and PA).	Girls and boys ages 12–14 (n = 92); Primarily white.	Discourse analysis; Focus groups presenting scenarios representing body-based harassment.	Found body-based harassment contributes to body image and relates to PA and nutrition. Noted boys took scenarios less seriously than girls: trivializing.
Klomsten AT, Marsh HW, Skaalvik EM 2005; Norway	To examine boys' and girls' perceptions of feminine/ masculine characteristics within sport and PE.	High school students (n = 357), unspecified ethnicity.	School-based questionnaires/scales (appearance, masculine and feminine traits).	Found boys and girls appear stereotyped in sport, in regard to masculine and feminine values.
Larkin J, Rice C 2005; Canada	To determine the extent to which curricula reflect body image concerns and make recommendations accordingly.	Girls in 7th-8th (n = 45) from 4 schools; diverse population.	Discourse analysis of Ontario health curricula; Workshops; Interviews.	Noted limitations of curricula: sending contradictory messages, not addressing school environment. Found body-based harassment played major role in constructing girls' bodies as problematic.
Lopez V, Corona R, Halfond R 2013; USA	To examine relationship between media influences, disordered eating, appearance concerns, and gender role orientation.	Adolescents (n = 96) with mean age 15.4, 58% female, in Latino community.	Survey (demographics, BMI, hating habits, appearance, culture, media, gender roles).	Found disordered eating associated with BMI, sociocultural values, and body ideals; Media influences associated with gender role orientation.
Mooney E, Farley H, Strugnell C 2009; Ireland	To investigate body image satisfaction and dieting in adolescent females.	Female adolescents age 15–16 (n = 124); unspecified ethnicity.	Qualitative semi-structured focus groups.	Found participants vulnerable to cultural demands, pressures of thinness, an body dissatisfaction.
Pesa JA, Syre TR, Jones E 2000; USA	To determine whether overweight female adolescents differ from normal weight peers in psychosocial factors while adjusting for body image.	Female adolescents in 7th-12th grades (n = 3197).	Part of National Longitudinal Study of Adolescent Health. Scale (depression, self-esteem, connectedness, etc.); Self-reported BMI.	Found, after controlling for body image, depression not a facet in differentiating overweight adolescent girls from peers. Noted relationship between low self-esteem and being overweight.
Richman EL, Shaffer DR 2000; USA	To explore relationship between female adolescents' precollege sport participation and college self-esteem.	Undergraduate students, (n = 220), mean age 19; 85% White.	Questionnaire (sport participation, social acceptance, academics, physical competence, body image, gender role, self-esteem).	Found early sport participation has positive impact on self esteem and body image, Noted sports promote self worth and foster flexible attitudes toward gender identity.
Robbins LB, Pender NJ, Kazanis AS 2003; USA	To identify barriers to PA reported by middle school girls.	Girls (n = 77) ages 11–14; diverse population.	Questionnaire based on trans-theoretical model (PA barriers).	Noted barriers with highest scores: Self-conscious, not motivated, lacking peers to take part with, too busy, bad day/tired, weather, etc.
Slater A, Tiggemann M 2010; Australia	To explore girls' reasons for ceasing participation in PA.	49 Girls in 8th-9th grades (n = 49), primarily White.	Focus groups.	Found that PA not being 'cool' or feminine was perceived as a major barrier for girls’ participation.
Vu MB, Murrie D, Gonzalez V, Jobe JB 2006; USA	To explore similarities and differences in girls' and boys' perceptions of girls' PA.	Girls (n = 180) and boys (n = 77) from 7th-8th grades, diverse population.	Part of TAAG intervention study; Focus groups separately with girls and boys, semi-structured interviews with girls only.	Found social influences important, and supportive environments required to support girls in PA.
Wetton AR, Radley R, Jones AR, Pearce MS 2013; UK	To understand reasons for girls not participating in extracurricular PA by exploring barriers.	Girls from 2 high schools (n = 60), ages 15–16, unspecified ethnicity.	Explorative mixed methodology. Questionnaire (availability of PA/ barriers); Individual semi-structured interviews.	Noted girls perceived lack of ability to engage in PA; 70% felt girls teams weren't treated equally by teachers. Participants perceived stereotypes in gender roles.
Whitehead S, Biddle S 2008; UK	To comprehensively explore adolescents girls and PA.	Girls ages 14–16 (n = 46); predominately white	Focus groups.	Found notions of femininity and achieving feminine ideals important; Suggested activity should be fun, informal, unstructured.
Witmer L, Bocarro JN, Henderson K 2011; USA	To explore adolescent girls' attitudes toward PA, eating, and health.	Girls (n = 28) in 6th-8th grades; diverse population.	Grounded theory approach; focus groups.	Found behavior and environment interrelated, adolescents strongly influenced by social norms and peers; Noted perceptions of gender appropriate behavior and fewer opportunities for girls.

**Table 3:** **Quantitative study characteristics**

**Article type**	**#**	**Participant age**	**#**	**Year published**	**#**	**Focus**	**#**	**Average sample size**	**#**
Qualitative	13	Early adolescent	3	1999-2003	9	PA only	15	Qualitative	65
Quantitative	7	Early/middle	6	2004-2008	11	Nutrition	5	Quantitative	1312
Mixed method	5	Mid adolescent	11	2009-2014	8	Both	8	Mixed method	44
Book chapter	1	Middle/late	2	**Study Description**	**#**	**Sex**	**#**	**Setting**	**#**
Commentary	1	Late adolescent	1	Intervention	3	Female only	19	School	21
Review	1	Teacher included	2	Part of larger study	6	Mixed	6	Community	4

### Theory, methodologies, and procedures

A variety of methodologies and procedures were described in the reviewed articles. The majority (11) of studies that included quantitative data collection used some form of survey or scale. Two studies measured anthropometrics or body composition, and one used accelerometers. The majority of these employed descriptive statistics, while several included multivariate or regression analyses, and two used path analysis to examine relationships between variables. Of the studies using qualitative techniques, eight used focus groups, six used interviews, one used collages and drawings, one used audio taped conversation, and one used curricula review. In regard to theory and methodology, six studies used discourse analysis or discursive theory, two of which paired discourse analysis with feminist post-structuralism, allowing dominant discourses that are institutionally reproduced to be analyzed and challenged [12,13]. Other studies employed grounded theory, social cognitive theory, interpretive description, reflexive description, and biographical analysis.

Some qualitative studies incorporated theories unique to this area. One used “the new social studies of childhood”, which regards children as active social agents and offers a method of understanding their experiences [14]. Another incorporated objectification theory, which explains how body-targeted harassment contributes to body satisfaction, self-consciousness, and feelings of fear or powerlessness [15]. Finally, a study referring to feminist theorist Foucault, incorporated corporeal femininities, which considers the importance of the body in discourse and the relationship between body, gender, and identities [16]. The same study incorporated performance theory and inhibited intentionality, which theorize about the ways girls behave in negotiation with dominant gender discourses, and the tendency of girls to underestimate their athletic ability and therefore inhibit their PA performance [16].

### Major themes

Five themes were identified through conducting thematic analysis of the data extracted from the articles reviewed. First, girls’ relationships with PA are complex and require complex negotiations of gender roles. Second, the literature appeared to focus on dieting or unhealthy weight control behaviors, rather than healthy nutrition. Third, appearance and perceptions influence girls’ nutrition and PA behaviors. Fourth, “body” focused discourse is significant to girls’ experiences with nutrition and PA. Finally, social influences, institutions, and environments are influential for girls, and may offer opportunity for future research, intervention, programming, and policy change.

### Girls’ relationships with PA are complex: negotiation of gender roles

Reviewed studies primarily centered on PA rather than nutrition, and noted the complexity of girls’ relationships with PA. Studies indicated that young girls enjoy PA and that it offers enhanced self-esteem, social benefits, health, and satisfaction, provides a creative outlet, and allows girls to feel proud [13,14,17-19]. The complexity, however, arises in consideration of the idea of femininity. Girls experience complex relationships with PA, in that they feel pressure to appear feminine and act accordingly, limiting their ability to behave outside the normal confines of heterosexual femininity [18,20-22]. Some girls may challenge these norms, but risk being perceived as overly masculine, resulting in what Cockburn and Clarke (2002) call a “femininity deficit” [12,13,16,20,23,24]. Girls may also perceive pressure to be both feminine and athletic, resulting in PA requiring complex negotiation of contradictory and ambiguous institutionalized gender discourse [12,13,16,20,23,24].

Gender stereotyping is prevalent and influential in girls’ PA. A study describing how youth spent their time indicated that boys spent time being active, while girls spent time socializing, while another indicated boys engaged more in soccer and hockey, while girls took part in dance and gymnastics [25,26]. Others indicated that girls recognized male dominance in sport, perceived sport to be less “cool” for girls, and felt teachers treated girls’ teams unequally [13,27]. In other cases, despite girls’ theoretical rejection of gender norms and support for equality, they described some activities as “too girlie”, and identified “boy” sports [12,14]. Interestingly, Richman and Shaffer [28] noted that early sport participation contributed to more flexible later attitudes toward gender identities. In general, qualities encouraged in PA, such as competitiveness and strength, oppose stereotypical feminine ideals. The complexity of these issues is evident, contributes to girls’ experiences with PA, and indicates the need for challenging sociocultural norms and existing double standards [12,20].

### Focus on dieting rather than nutrition

In the studies reviewed, the nutrition-centered literature was predominately focused on unhealthy weight control behaviors rather than healthy nutrition (such as promotion of fruits and vegetables). Of the studies focused beyond dieting, an intervention aimed at both nutrition and PA, noted minimal change in eating attitudes and behaviors and suggested the intervention required further refinement, though did not specify how this refinement might occur [29]. In a review, DeRose, Das, and Millman [30] noted that while some research may indicate females have better nutritional habits than males, their results were difficult to interpret, and should not be perceived as reason to discontinue attempting to raise the status of women and girls. Finally, Witmer et al., [22] discussed how families promoted healthy eating, but also encouraged indulgences such as movie nights and holidays that were associated with unhealthy foods, indicating important and complex social influences on nutrition.

Dieting and body size were frequently noted in the review. Dunkley, Wertheim, and Paxton (2001) noted only 15% of participants had never watched their weight, while nearly 70% of normal-weight participants had an ideal weight less than their current. Several studies indicated that dieting and weight loss behaviors were common for young girls [13,23,31,32]. Others reported young girls engaging in behaviors such as fasting and meal skipping as a result of media, sociocultural norms, body ideals, and misconceptions about food [31-34]. Larkin and Rice [35], examined school curricula and found that the “healthy eating, healthy weights” discourse sent contradictory messages by instructing students to eat well, but also to accept their bodies, irrespective of weight status. These lessons ignore the complexity underlying nutrition, and indicate the challenge of instructing nutrition without causing disordered eating, in a broader environment that promotes poor nutritional behaviors [35].

### Appearance and perceptions influence behaviors

A significant theme was that young girls are concerned about appearance and how others may perceive them, which was emphasized in the PA literature. Several studies indicated that adolescent girls were uncomfortable engaging in PA, and physical education (PE), because they were pressured to wear athletic attire or uniforms, wanted to avoid getting dirty or sweaty, and were not given sufficient time following PA for showering or applying makeup, circumstances which made them feel self-conscious, uncomfortable, and vulnerable [13,19-21,36,37]. The importance of being perceived within a constrained image of not only thin, but also “toned” or athletic, was noted, and found to contribute to behavioral adjustments in both PA and nutrition [23].

The complex negotiation of gender roles (previously described in the first theme above) also relate to appearance and perceptions. Girls in the reviewed studies stressed the importance of being perceived by their peers as engaging in socially constructed gender appropriate behaviors and achieving feminine ideals [19,21,22]. While some girls wanted to be perceived as strong or capable, there was a noted need to balance those qualities to avoid being perceived as muscular or aggressive, or alternatively, being perceived as lazy or overweight [21,37]. Studies also noted that girls discussed modifying participation in PA by taking part in large group activities in hopes of going unnoticed, or through inhibited intentionality, the theory that girls may underestimate their ability and underperform accordingly [16,18]. The presence of male gaze and fear of teasing or inadequacy also added to PA discomfort, with authors of one study noting only 20% of girls who lacked confidence felt comfortable in PE class [16].

### “Body” focused discourse is significant to Girls’ experiences

Reviewed articles included discussion of the female body, particularly in regard to socially constructed ideals. One author noted girls did not discuss health as a priority and defined it in relation to body size [22]. In another study, while boys rated strength and masculinity as important, girls indicated appearance, particularly being “slender” as important, and described ideal bodies as “thin” and “pretty” [26]. Dunkley et al. [32] indicated that body dissatisfaction was contributed to by media, parents, and peers, but was best predicted by measured larger body size. A quantitative study noted that without the influence of body image, depression did not contribute to differentiating overweight girls from their peers [38]. Young girls are vulnerable to unattainable ideals, and their complex relationships with their bodies potentially help reproduce them [23,24,31]. Body-centered discourse was furthered through discussion of body-based harassment, which contributes to disordered eating and constructing girls’ bodies as problematic [35]. In a study that presented harassment scenarios, adolescents indicated that body-based harassment may make girls embarrassed or self-conscious, and may result in withdrawing from PA or restricting eating [15]. Interestingly, researchers also noted that some girls perceived certain messages as flattering, and that male participants had a tendency to trivialize scenarios [15]. That study highlighted the societal complexities associated with female bodies and, that body-based harassment relates to body image, PA, and nutrition [15].

Several studies incorporated discussion of media influences. One noted the importance of body image and how participants were influenced by celebrities, by even being aware of their diet and PA routines, concluding that media promotes thin ideals [31]. Other studies explored the relationship between media and gender role orientation, noting media influences body ideals and dieting, and suggesting the need to reduce favoritism toward male athletes [27,32,34]. Finally, a commentary examining media’s influence on body image indicated that women have historically been challenged by unattainable ideals associated with femininity, and that current media portrays mixed messaged about what is feminine and attractive [39]. It is clear that the media promotes body ideals, especially in regard to socially constructed femininity, and as some authors suggest, may indicate an opportunity for intervention [39].

### Social influences, institutions, and environment

The importance of social influences, institutions, and environments (schools, for example) in relation to young girls’ PA and nutrition was evident from the review, and was often cited as a means of potential improvement or action. Brooks and Magnusson [14] highlighted the importance of fun, and that girls need a PA environment focused on encouragement rather than athletic ability. Other studies highlighted the impact of teachers’ attitudes and expectations, especially in PE, and the importance of creating an environment that supports gender equality and builds confidence in girls’ abilities [13,17]. Finally, others described the importance of shifting norms and creating supportive environments, though did not offer concrete or specific examples of how to accomplish this [33,37].

Some studies noted societal level influences, calling for a redefinition of gender roles, body image to be discussed beyond the health classroom, and the requirement for enhanced tactics to battle societal messaging toward young girls [24,35,38]. Other studies noted the many factors that influence young girls and suggested that interventions need to address multiple factors and agents, such as peers, teachers, parents, and media [13,32]. Finally, some articles suggested that environments need to be created where girls can receive education and resist, question, and negotiate those experiences in empowering ways, a responsibility that should be considered for programmers, teachers, and policy makers [16,18]. In sum, broad system and societal changes are clearly required, though the literature lacks practical ways to accomplish these goals.

## Discussion and conclusions

Gender norms, ideals, and stereotypes clearly influence young girls’ PA and nutrition. The themes discussed above detail the relationships young girls have with gender norms, PA, and nutrition. First, girls’ relationships with PA require incredibly complex negotiations of gender roles, and given this complexity, it is not surprising that adolescent girls become less active as they develop. Second, the literature was focused on dieting and unhealthy weight control behaviors, rather than healthy nutrition. This focus suggests a gap from a population health perspective, in that discourse focused on unhealthy behavior continues to problematize behavior of individuals, rather than creating supportive environments for all. Third, young girls are concerned about appearance and influenced by how they may be perceived (by their peers, social groups, teachers, and families), which speaks to how socially acceptable norms influence healthy eating and PA. Fourth, current popular and academic discourse focuses on the body of young girls, and this is significant to girls’ experiences with nutrition and PA. Finally, institutions, environments, and social norms provide high-level influences on girls’ PA and nutrition and may offer opportunity for future research and action.

It is clear that these themes, while offering a way to organize the current literature, are not independent of one another. Elements of body-focused discourse (discussed in the fourth theme) are evident in the first two themes describing negotiation of gender roles in PA and unhealthy weight control behaviors. The complex negotiations of gender roles described in the first theme are evident in all of the other themes. Common unhealthy weight control behaviors are clearly influenced by the final theme relating to social institutions and environments. The third theme (discussing appearance and perceptions), and the fourth (body-focused discourse) are intricately intertwined. While the theme discussing appearance was highlighted by the PA literature, it is connected to dieting and nutrition as well. The themes described by this paper intersect, overlap, and blend together, further contributing to the complexity of the subject, and suggesting future research should consider these intersections.

This review indicates several gaps in the literature. First, reviewed studies focused on early-middle adolescence and very few focused on late adolescence, i.e., ages 16–19 years (as noted in Table 3). This is common in much of the PA and nutrition literature where studies focus on the elementary school environment. This offers an interesting opportunity for future research, as girls in the older age group may be more aware of socially constructed ideals and more able to understand societal norms and influences. In regard to methodology, a majority of reviewed articles had a qualitative focus, which is understandable given the subject matter, though a mixed methods study might offer valuable insight in regard to both health outcomes and experiences of young girls. The qualitative studies in this review also incorporated a unique mix of theory and methodology. Finally, while the value of using socio-ecological frameworks to understand multiple levels of environmental influences on health behaviors is well established, only one quantitative study in this review incorporated this model to explore factors associated with PE enjoyment, offering opportunity for future research [17].

The intention of this review was to examine the academic literature on how gender norms are understood in young girls’ PA *and* nutrition. Of the 28 documents reviewed, only 8 actually focused on both. Further, there was a substantial focus on PA, rather than nutrition, and as mentioned above, those looking at nutrition focused on dieting and disordered eating. Future research should examine both PA and nutrition, and should focus on gender norms in relation to young girls’ healthy nutrition habits, rather than unhealthy eating practices. The current public health discourse that focuses on obesity in youth problematizes the bodies of young people. Researchers, policy makers, and intervention planners and implementers need to be aware of furthering discourse that potentially contributes to this issue, and aim rather to understand and deconstruct this discourse in a manner that is empowering, engaging, and healthy, specifically for young girls (though the influence of this discourse on young boys is beyond the scope of this study). This would mean shifting the focus away from obesity as a health concern toward encouragement of healthy behaviors, focusing on gender equality, and engaging youth in research and policy-making.

Strengths of this review include the unique focus on gender norms, young girls, PA, and nutrition, with a health promotion perspective, the expansive inter-disciplinary search strategy and broad range of studies included for review in order to summarize the current literature. One limitation of the review was that, in order to locate articles related to both PA and nutrition, the ‘AND’ search function was used; as many articles focusing on one or the other were still retrieved, it is likely that other articles focused on only PA or nutrition were excluded as a result. A potential limitation of scoping studies is that due to their broad nature and heterogeneity of included results, quality assessment is not typically part of the review.

In conclusion, young girls’ PA and nutrition is affected by gender norms and feminine ideals through complex negotiations, perceptions, body-centered discourse, and societal influences. Future research should focus on older adolescents, employ mixed methods and the social ecological model in conjunction with gender-specific theory, and explore both PA and nutrition, focusing on health eating rather than unhealthy weight control behaviors.

## Abbreviations

PA: Physical activity
PE: Physical education
